# Pediatric Colonoscopy in South China: A 12-Year Experience in a Tertiary Center

**DOI:** 10.1371/journal.pone.0095933

**Published:** 2014-04-23

**Authors:** Pingguang Lei, Fang Gu, Liru Hong, Yuli Sun, Minrui Li, Huiling Wang, Bihui Zhong, Minhu Chen, Yi Cui, Shenghong Zhang

**Affiliations:** 1 Division of Gastroenterology, Shenzhen Bao'an District Songgang People's Hospital, Shenzhen, China; 2 Division of Gastroenterology, The First Affiliated Hospital, Sun Yat-sen University, Guangzhou, China; 3 Division of Reproductive Medicine, The First Affiliated Hospital, Sun Yat-sen University, Guangzhou, China; 4 Division of Gastroenterology, Affiliated Shantou Hospital of Sun Yat-sen University, Shantou, China; University of Aberdeen, United Kingdom

## Abstract

**Objective:**

To investigate: 1) the demographics and clinical characteristics, 2) the findings, and 3) the safety and effectiveness in a cohort of Chinese pediatric patients undergoing colonoscopy.

**Methods:**

The study participants were consecutive patients aged ≤14 years old that underwent their first colonoscopy in the endoscopy center at the First Affiliated Hospital, Sun Yat-sen University between Jan. 1, 2001 and Dec. 31, 2012. Demographic, clinical, endoscopic, and pathological findings were collected.

**Results:**

The cohort consisted of 322 patients, including 218 boys (67.7%) and 104 girls (32.3%). The median age was 8.0 years old and ranged from 9 months to 14 years old. Hematochezia (48.8%) and abdominal pain/discomfort (41.3%) were the most common presentations preceding pediatric colonoscopy. The caecal intubation success rate was 96.3%. No serious complications occurred during the procedures. A total of 227 patients (70.5%) received a positive diagnosis under endoscopy, including 138 patients with polyps and 53 patients with inflammatory bowel disease (IBD). Among the patients with polyps, 71.0% were juvenile polyps. Comparisons between years 2001–2006 and 2007–2012 showed that the IBD detection rate increased significantly (4.6% *vs*. 22.4%, *P*<0.001), while the opposite occurred for the polyp detection rate (73.1% *vs*. 27.6%, *P*<0.001).

**Conclusion:**

Colonoscopy in pediatric patients is a safe and effective procedure. Polyps are the primary finding during colonoscopy. In South China there has been an increase in pediatric patients diagnosed with IBD over the past decade. However, a large epidemiological study is needed to confirm our findings.

## Introduction

Since its introduction in the late 1970s, the colonoscopy field has developed rapidly, especially in the 21^st^ century [1], [2]. Colonoscopies are now widely and routinely performed worldwide, both as a diagnostic and a therapeutic tool. The safety and effectiveness of diagnostic or therapeutic colonoscopies in adults has been well established in the past two decades [1], [3]. However, its utility for pediatric patients is only recently emerging. Major hurdles for pediatric colonoscopy include the high level of technical challenge, poor compliance with bowel cleansing, and uncooperativeness during the procedure. The slightly higher risk of severe complications when compared with adults might also restrict its use in pediatric patients [1].

The indications for colonoscopy in pediatric patients are similar to adult patients. Hematochezia, unrelieved abdominal pain, and unexplained diarrhea are the most frequent symptoms [1], [3]. Colorectal polyps are common during childhood and usually present with painless colorectal bleeding. The juvenile polyp is the most common type of colorectal polyp and is generally considered benign [1]. However, recent studies have reported cases of adenomatous change in juvenile polyps, indicating their neoplastic potential [4], [5]. Furthermore the incidence of inflammatory bowel disease (IBD) is increasing worldwide. It has been reported that approximately 20–30% of IBD patients are diagnosed in childhood [6]. Early detection of both polyps and IBD can significantly improve the patients' quality of life and overall health. Therefore, in pediatric patients, colonoscopy is the best tool both for early lesion detection and as an effective therapy to treat lesions and remove polyps.

Currently there are some retrospective reports about the utility of colonoscopy in pediatric patients [7]–[10]. However, data regarding the clinical features, endoscopic findings, and the safety and efficacy of pediatric colonoscopy are limited, especially in China. In view of this, we conducted a retrospective cohort study on the Chinese population. The aims of the study were to investigate: 1) the demographics and clinical characteristics, 2) the findings, and 3) the safety and effectiveness in a cohort of pediatric patients undergoing colonoscopy.

## Patients and Methods

### Ethics statement

This study protocol was approved by the Human Ethics Committee of The First Affiliated Hospital, Sun Yat-sen University and was conducted according to Declaration of Helsinki. Written consent was given by the patients' legal guardians for their information to be stored in the hospital database and used for research. All the data were stored at our endoscopy center, and were available to other scientists.

### Study protocol and data collection

**Figure 1:**
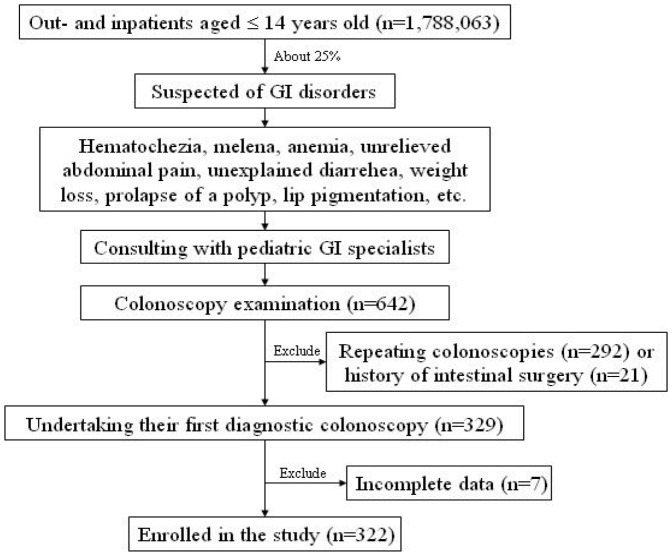
Management protocol for this study. Children aged ≤14 years old undertaking their first diagnostic colonoscopy at the endoscopy center of the First Affiliated Hospital, Sun Yat-sen University between Jan. 1, 2001 and Dec. 31, 2012 were enrolled in the study.

Children were referred for colonoscopy at the endoscopy center of the First Affiliated Hospital, Sun Yat-sen University, after a comprehensive evaluation by a pediatric gastroenterologist. The management protocol for the study is summarized in **Figure 1**. For this study, we retrospectively reviewed the medical records of consecutive pediatric patients aged ≤14 years old undertaking their first diagnostic colonoscopy between Jan. 1, 2001 and Dec. 31, 2012.

Endoscopic procedures were performed by four experienced specialists. Prof. Cui performed approximately 180 procedures, and the other doctors performed approximately 50 procedures each, on average. The bowel cleansing preparation protocol for colonoscopy in pediatric patients varied depending on the patients' age, weight, clinical state, and cooperation. Infants were restricted to the ingestion of clear liquids for approximately 12–24 hours prior to the procedure and a saline solution enema. Older pediatric patients were treated with laxatives containing Fleet phospho-Soda or polyethyleneglycol, and in most cases this was effective and satisfactory. The method of sedation or anesthesia was selected at the discretion of the endoscopists and anesthetists. The most common anesthesia was propofol or ketamine, and the most common sedation was a combination of midazolam and pethidine. Patients' vital signs were monitored throughout the procedure. Endoscopes were selected based on the age and body weight of the patients. The gastroscopes used in adult patients could be used in pediatric patients above approximately 10 Kilograms. Smaller endoscopes are appropriate for smaller children. Colonoscopes used in adult patients can be used in patients above 10 years old. Mild complications related to the procedure included abdominal bloating (for >1 hour after the procedure), minimal or mild bleeding (<50 ml), mucosal injury (confined to the superficial mucosa), transient hypotension (a decrease in blood pressure >30 mmHg for <1 min) and hypoxemia (SaPO_2_<90% for <1 min). Serious complications included accidental anesthetic death, massive bleeding (>50 ml), perforation, and bacteremia.

Records included the patient's gender, age, clinical presentations for colonoscopy referral, the pathological findings and diagnoses resulting from the colonoscopy, the procedural success rate, and complications during and following the procedure. During caecal intubation, the endoscope should probe to the caecum, while ileal intubation should probe the terminal ileum. The rates for these procedures were calculated independently. The mucosal biopsy depended on ultimate diagnosis and the specialists' experience. Biopsy was not routinely performed if nothing abnormal was found macroscopically, except for those highly suspected of eosinophilic colitis based on clinical manifestations and peripheral eosinophilia. Patients were divided into two groups based on the date of their colonoscopy with the first period including 2001–2006 and the second period including 2007–2012. Comparisons of gender, age, findings, and diagnoses resulting from the colonoscopy were performed between these two groups.

### Statistical Analyses

Statistical analyses were performed using the SPSS software (version 16.0; SPSS, Inc., Chicago, IL). The results are reported with the median age or the percent frequency for the categorical data. The demographics and findings under colonoscopy were compared between the two chronological periods (2001–2006 and 2007–2012). Continuous variables were tabulated using the Mann-Whitney U test for non-normal data distribution and the Student t test for normal data distribution. Categorical variables were analyzed with a chi-squared test. All hypotheses were two-tailed and statistical significance was determined by a *P* value less than 0.05.

## Results

### Demographic and clinical characteristics

Between Jan. 1, 2001 and Dec. 31, 2012, approximately 31,712,045 outpatients and 595,618 inpatients were treated at the First Affiliated Hospital, Sun Yat-sen University. Among these, the number of pediatric outpatients and inpatients, aged ≤14 years old, was 1,746,194 and 41,869, respectively. In our endoscopy center, 57,246 patients underwent colonoscopy and 115,327 patients (1013 pediatric patients) received upper gastrointestinal endoscopy examination during this period. A total of 322 pediatric patients (0.54%) undergoing colonoscopy were included in the study; 218 boys (67.7%) and 104 girls (32.3%) with a male to female ratio of 2.1∶1. Among these pediatric patients, only 47 patients (14.6%) also received upper endoscopy examination. The median age was 8.0 years old with a range of 9 months to 14.0 years old. These results were similar when comparing boys and girls (8.0 *vs*. 8.0 years old, *P*>0.05). Hematochezia, abdominal pain/discomfort, and diarrhea were the most common presentations for a pediatric endoscopy referral, accounting for 157 (48.8%), 133 (41.3%), and 38 (11.8%) patients, respectively. Other presentations included fever (n = 21, 6.5%), altered fecal characteristics (n = 18, 5.6%), anemia (n = 15, 4.7%), change in bowel habits (n = 12, 3.7%), weight loss (n = 11, 3.4%), polyp prolapse from the anus (n = 8, 2.5%), vomiting (n = 7, 2.2%), lip pigmentation (n = 6, 1.9%), and others (n = 3, 0.9%).

### Findings under colonoscopy

A total of 227 patients (70.5%) had a positive diagnosis under colonoscopy, while 95 patients (29.5%) did not show any abnormality. Polyps (n = 138, 42.9%) and IBD (n = 53, 16.5%) were the two most common positive diagnoses. Additional findings included nonspecific enteritis (n = 29, 9.0%), colonic cancer (n = 1, 0.3%), and others (n = 6, 1.9%). Hematochezia (n = 113, 81.9%) was the most common symptom in pediatric patients with polyps, while abdominal pain was the most common in patients with IBD (n = 41, 77.4%). Among the polyp patients, 95 were boys (68.8%) and 43 were girls (31.2%). The majority of the polyps were juvenile (n = 98, 71.0%) and solitary (n = 111, 80.4%). The rectum (n = 88, 63.8%) and sigmoid colon (n = 38, 27.5%) were the most common sites for polyps, followed by the descending colon (n = 20, 14.5%), transverse colon (n = 18, 13.0%), and ascending colon (n = 11, 8.0%). Occasionally, polyps were located at the caecum colon (n = 4, 2.9%), hepatic flexure (n = 3, 2.2%), and splenic flexure (n = 1, 0.7%). Among patients with IBD, Crohn's disease (CD) (n = 45, 84.9%) was more common than ulcerative colitis (UC) (n = 8, 15.0%). Two cases of IBD-unclassified (IBD-U) were diagnosed at first. However, during the follow up, they were then diagnosed as CD. The detection rate of granuloma in CD was about 35.6% (16/45).

### Safety and effectiveness of colonoscopy

The majority of patients (n = 237, 73.6%) were anesthetized with propofol. Fifty-one patients (15.8%) received ketamine. A combination of midazolam and pethidine was used to sedate 34 patients (10.6%). During the procedure hypoxemia occurred in 13 patients (4.0%) and hypotension occurred in 5 patients (1.6%). All the patients recovered following the procedure. The caecal intubation success rate was 96.3%, and only 12 procedures failed to reach the caecum. Among the failed cases, one had an adenocarcinoma in the ascending colon, hindering endoscopic passage. In nine cases the procedure was stopped due to hypotension or hypoxemia. Another two procedures failed due to narrow cavities, possibly the result of intestinal adhesion, because they had a history of surgery in the kidney or ureter. Whether the patients needed ileal intubation was determined by our experienced specialists. Usually in patients especially in those suspected of IBD or nothing abnormal found in the colorectum, ileal intubation was routinely performed. Attempts to ileal intubation were performed in 268 patients, and the success rate was 81.7%. Except 12 failed cases in caecal intubation, another 37 procedures failed to reach the ileum by multiple attempts, mainly due to the narrow ileocecal valve (n = 11) or loops formation during the procedure (n = 26).

Overall, mild complications occurred in only 29 patients (9.0%). No serious complications, such as massive bleeding or perforation, occurred during the procedure. The number of biopsies depended on ultimate diagnosis. They were usually taken from the located lesions. In IBD patients, multiple biopsies (3–5 on average) were routinely performed in each inflamed lesion, while in patients with polyps, especially single polyps with small size, fewer biopsies were needed (1–2 on average). Pediatric patients who had a colonic polypectomy or biopsy were admitted for observation of colonic bleeding at least overnight. No patients were readmitted to the hospital for complications following the colonoscopy procedure. In a majority of patients (94.9%), the polyps could be removed on the first attempt without bleeding or perforation. Seven patients had repeated colonic polypectomies due to multiple polyps throughout the colon.

### Comparisons between 2001–2006 and 2007–2012

**Figure 2:**
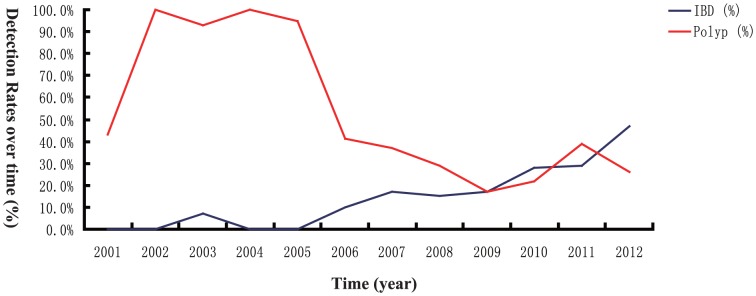
Changes on the detection rates of polyp and IBD over time. The detection rates of polyp and IBD in pediatric patients were analyzed year by year and compared over time (2001–2012).

The polyp detection rate decreased, while IBD detection rate increased over time during the past 12 years (**Figure 2**). Comparisons between the two time periods (2001–2006 and 2007–2012) indicated that the median patient age was increasing (5.0 *vs*. 9.0 years old, *P* = 0.002), with the male to female ratio remaining similar (2 *vs*. 2.1, *P* = 0.778). There was also a statistically significant trend towards a higher IBD diagnosis rate (4.6% *vs*. 22.4%, χ^2^ = 16.539, *P*<0.001), while the opposite was true for polyps (73.1% *vs*. 27.6%, χ^2^ = 60.885, *P*<0.001) (Table 1). In the polyp patients, the gender ratio and age were similar between the two periods (*P*>0.05).

**Table 1:** Comparisons of gender, age, and colonoscopic findings between the years 2001–2006 and 2007–2012.

	2001–2006 (n = 108)	2007–2012 (n = 214)	*P* value
Gender			
Male, n (%)	72 (66.7)	146 (68.2)	0.778
Female, n (%)	36 (33.3)	68 (31.8)	
Median age in years (IQR^a^)	5.0 (3.1–10.6)	9.0 (4.5–13.0)	0.002
Colonoscopic findings			
IBD^b^, n (%)	5 (4.6)	48 (22.4)	<0.001
Polyp, n (%)	79 (73.1)	59 (27.6)	<0.001

IQR^a^: interquartile range; IBD^b^: inflammatory bowel disease.

## Discussion

In this study, we investigated the epidemiological profile of the pediatric patients who underwent colonoscopy and the safety of this procedure. Polyps and IBD were the most common diagnoses and colonoscopy was generally a safe and effective diagnostic and therapeutic procedure in pediatric patients.

In this study, we defined the age criteria for pediatric patients as ≤14 years old, primarily based on the WHO age classification [11]. Various cut-offs ranging from 16 to 20 years old have been used in previous studies [6]–[10]. The median age in our study was 8.0 years old, and was similar to that in several previous reports [1], [12], but older than that reported in other studies [7], [13]. The male to female ratio in this study was 2.1∶1, which was consistent with previous studies [1], [10], [12]. However, the reason for this gender bias is not well understood thus far.

Over the last few decades, pediatric colonoscopy has been widely performed for various clinical applications, including the evaluation of gastrointestinal bleeding, IBD, or radiographic abnormalities, and for pathological tissue diagnosis. The safety of pediatric colonoscopy has been established, although the immediate complication rate of pediatric colonoscopy has been reported to be 1.1%, which is higher than that in adults (0.3%) [14], [15]. Our study showed no serious complications during the procedure, such as bleeding and perforation. The risk of severe complications could be reduced significantly with conscientious procedure performance, thorough bowel cleansing, sufficient risk evaluation preceding the procedure, and appropriate anesthesia or sedation.

Due to the slightly higher risk of complications in children, indications for pediatric colonoscopy are usually more stringent than for adults. Hematochezia, potential IBD, or cancer surveillance are reported to be the most common indications for the procedure [1]. In our study, hematochezia, abdominal pain/discomfort, and diarrhea were the most common presentations for pediatric endoscopy referral. Hematochezia was the most common symptom for patients with polyps, while abdominal pain was the most common in IBD patients. These findings were similar to previous studies [1], [7], [9], [12]. For pediatric patients presenting with recurrent hematochezia, unrelieved abdominal pain, or unexplained diarrhea, colonoscopy may be the most useful diagnostic tool.

Compared to adults, pediatric patients had a higher frequency of positive findings resulting from colonoscopy [7], [10]. In our study, 70.5% of the patients had a positive diagnosis. Colorectal polyps were the most common findings in children, with a reported incidence of 1% [16]. No epidemiological data regarding the overall incidence of colorectal polyps in China are currently available. In the literature, the prevalence of colorectal polyps varied greatly in pediatric patients who underwent colonoscopy. In the US, a large cohort from 14 medical centers reported that colorectal polyps were detected in 6.1% of pediatric patients undergoing colonoscopy [10]. In another study conducted in Hong Kong, the detection rate for pediatric patients was 29.1% [7]. In our study, colorectal polyps were the most prevalent finding and were identified in 42.9% of the patients. The variation in detection rate might be due to the difference in colonoscopy indications within each healthcare system, as well as the patients' age and ethnicity. Juvenile polyps are the most common type of polyp and the majorities are located in the rectum and sigmoid colo [4], [7], [9], [13], [17]–[22]. Our results are consistent with these previous findings. However, polyps located in the proximal colon were not uncommon; moreover, previous studies suggest that the detection rate of polyps in the proximal colon is increasing [1], [10]. Therefore, the use of pancolonoscopy in the evaluation of gastrointestinal pathology should be considered more frequently.

IBD, which mainly includes CD and UC, is an idiopathic recurrent gastrointestinal disorder associated with immunological dysfunction. In the present study, it was the second-most common positive finding in pediatric colonoscopy patients. IBD was once rare in Chinese patients and previously considered to be a “Western” disease. Resulting from dramatic economic development in the past two decades, dietary habits and life styles have gradually shifted towards a more western approach, especially in South China where the present study was conducted. The epidemiology of IBD has changed in many ways over the past decade. Community-based epidemiological studies, both by our group and the Asia-Pacific IBD study group, have shown a marked increase in the incidence of IBD in Asian adults [23], [24]. In pediatric patients, several studies also found an increasing incidence of pediatric IBD during the past decade [25], [26]. Our results suggested that a similar increase might also exist in pediatric patients in South China, with the IBD detection rate significantly higher during 2007–2012 than during 2001–2006. In our study, CD patients were more common than UC patients. This is in accordance with previous findings that the onset age in CD patients is much earlier than UC patients [6]. It was also reported that the incidence of pediatric UC has stabilized in some countries, while the incidence of pediatric CD continues to rise quickly in most regions [27], [28]. This is consistent with our findings that pediatric CD was more common than UC. Comparisons between the two groups indicated that the median patient age was increasing. This may be due to an increase in IBD patients during this time. The median age of IBD patients in the 2007–2012 period was 12.5 years old, much older than those with polyps (5.0 years old). IBD patients consistently presented with abdominal pain, hematochezia, or non-infected diarrhea. In order to diagnose this disease earlier, colonoscopy should be considered the preferred diagnostic tool. Early IBD detection would allow pediatric patients to be treated earlier and improve their overall prognosis.

## Conclusion

Our study found that colonoscopy in Chinese pediatric patients was safe and effective, both as a diagnostic and therapeutic procedure. Hematochezia and abdominal pain/discomfort were the most common presentations for pediatric endoscopy referral. Polyps were the most common finding during colonoscopy, followed by IBD. More pediatric IBD patients have been found over the past decade. However, there are several limitations in this study. First, the study is conducted retrospectively. Second, our data are collected from one single clinical center, although our hospital manages a large regional population. Therefore, a large prospective multi-center epidemiological study is needed to confirm these findings.

## References

[1] Park JH (2010) Role of colonoscopy in the diagnosis and treatment of pediatric lower gastrointestinal disorders. Korean J Pediatr 53: 824–829. 21189966 10.3345/kjp.2010.53.9.824 PMC3005213

[2] Victor LF (1996) Colonoscopy. In: Walker WA, Durie PR, Hamilton JR, Walker-Smith JA, WatkinsJB, editors Pediatric gastrointestinal disease. Pathophysiology, diagnosis, management. 2nd ed. St. Louis: Mosby 1533–1541.

[3] Friedt M, Welsch S (2013) An update on pediatric endoscopy. Eur J Med Res 18: 24. 23885793 10.1186/2047-783X-18-24 PMC3751043

[4] Poddar U, Thapa BR, Vaiphei K, Singh K (1998) Colonic polyps: experience of 236 Indian children. Am J Gastroenterol 93: 619–622. 9576458 10.1111/j.1572-0241.1998.176_b.x

[5] Fox VL, Perros S, Jiang H, Goldsmith JD (2010) Juvenile polyps: recurrence in patients with multiple and solitary polyps. Clin Gastroenterol Hepatol 8: 795–799. 20580940 10.1016/j.cgh.2010.05.010

[6] Lev-Tzion R, Turner D (2012) Is pediatric IBD treatment different than in adults? Minerva Gastroenterol Dietol 58: 137–150. 22643596

[7] Tam YH, Lee KH, Chan KW, Sihoe JD, Cheung ST, et al (2010) Colonoscopy in Hong Kong Chinese children. World J Gastroenterol 16: 1119–1122. 20205284 10.3748/wjg.v16.i9.1119 PMC2835790

[8] Mudawi HM, El Tahir MA, Suleiman SH, Eltaybe NH, Gamer NM, et al (2009) Paediatric gastrointestinal endoscopy: experience in a Sudanese university hospital. East Mediterr Health J 15: 1027–1031. 20187556

[9] Wei C, Dayong W, Liqun J, Xiaoman W, Yu W, et al (2012) Colorectal polyps in children: a retrospective study of clinical features and the value of ultrasonography in their diagnosis. J Pediatr Surg 47: 1853–1858. 23084197 10.1016/j.jpedsurg.2012.05.024

[10] Thakkar K, Alsarraj A, Fong E, Holub JL, Gilger MA, et al (2012) Prevalence of Colorectal Polyps in Pediatric Colonoscopy. Dig Dis Sci 57: 1050–1055. 22147243 10.1007/s10620-011-1972-8 PMC3878076

[11] Zhang SH, Cui Y, Weng ZJ, Gong XR, Chen MH, et al (2009) Changes on the disease pattern of primary colorectal cancers in Southern China: a retrospective study of 20 years. Int J Colorectal Dis 24: 943–949. 19424708 10.1007/s00384-009-0726-y

[12] Park KH, Park JH (2007) Changes in pediatric gastrointestinal endoscopy: review of a recent hospital experience. Korean J Pediatr Gastroenterol Nutr 10: 20–27.

[13] Haghi Ashtiani MT, Monajemzadeh M, Motamed F, Moradi Tabriz H, Mahjoub F, et al (2009) Colorectal polyps: a clinical, endoscopic and pathologic study in Iranian children. Med Princ Pract 18: 53–56. 19060492 10.1159/000163047

[14] Thakkar K, El-Serag HB, Mattek N, Gilger M (2008) Complications of pediatric colonoscopy: A five-year multicenter experience. Clin Gastroenterol Hepatol 6: 515–520. 18356115 10.1016/j.cgh.2008.01.007 PMC4999080

[15] ASGE Technology Committee, Barth BA, Banerjee S, Bhat YM, Desilets DJ, Gottlieb KT, et al (2012) Equipment for pediatric endoscopy. Gastrointest Endosc 76: 8–17. 22579260 10.1016/j.gie.2012.02.023

[16] Mestre JR (1986) The change pattern of juvenile polyps. Am J Gastroenterol 81: 312–314. 3706244

[17] Lee BG, Shin SH, Lee YA, Wi JH, Lee YJ, et al (2012) Juvenile polyp and colonoscopic polypectomy in childhood. Pediatr Gastroenterol Hepatol Nutr 15: 250–255. 24010095 10.5223/pghn.2012.15.4.250 PMC3746052

[18] Hood B, Bigler S, Bishop P, Liu H, Ahmad N, et al (2011) Juvenile Polyps and Juvenile Polyp Syndromes in Children: A Clinical and Endoscopic Survey. Clin Pediatr 50: 910–915. 10.1177/0009922811407177 21576185

[19] Ukarapol N, Singhavejakul J, Lertprasertsuk N, Wongsawasdi L (2007) Juvenile polyp in thai children-clinical and colonoscopic presentation. World J Surg 31: 395–398. 17235457 10.1007/s00268-005-0686-5

[20] Waitayakul S, Singhavejsakul J, Ukarapol N (2004) Clinical characteristics of colorectal polyp in Thai children: a retrospective study. J Med Assoc Thai 87: 41–46. 14971533

[21] Lee HJ, Lee JH, Lee JS, Choe YH (2010) Is Colonoscopy Necessary in Children Suspected of Having Colonic Polyps? Gut Liver 4: 326–331. 20981208 10.5009/gnl.2010.4.3.326 PMC2956343

[22] Thakkar K, Fishman DS, Gilger MA (2012) Colorectal polyps in childhood. Curr Opin Pediatr 24: 632–637. 22890064 10.1097/MOP.0b013e328357419f

[23] Zeng Z, Zhu Z, Yang Y, Ruan W, Peng X, et al (2013) Incidence and clinical characteristics of inflammatory bowel disease in a developed region of Guangdong Province, China: a prospective population-based study. J Gastroenterol Hepatol 28: 1148–1153. 23432198 10.1111/jgh.12164

[24] Ng SC, Tang W, Ching JY, Wong M, Chow CM, et al (2013) Incidence and Phenotype of Inflammatory Bowel Disease Based on Results From the Asia-Pacific Crohn's and Colitis Epidemiology Study. Gastroenterology 145: 158–165. 23583432 10.1053/j.gastro.2013.04.007

[25] Wang XQ, Zhang Y, Xu CD, Jiang LR, Huang Y, et al (2013) Inflammatory bowel disease in Chinese children: a multicenter analysis over a decade from Shanghai. Inflamm Bowel Dis 19: 423–428. 23340680 10.1097/MIB.0b013e318286f9f2

[26] Kim BJ, Song SM, Kim KM, Lee YJ, Rhee KW, et al (2010) Characteristics and Trends in the Incidence of Inflammatory Bowel Disease in Korean Children: A Single-Center Experience. Dig Dis Sci 55: 1989–1995. 19763825 10.1007/s10620-009-0963-5

[27] Benchimol EI, Fortinsky KJ, Gozdyra P, Van den Heuvel M, Van Limbergen J, et al (2011) Epidemiology of pediatric inflammatory bowel disease: a systematic review of international trends. Inflamm Bowel Dis 17: 423–439. 20564651 10.1002/ibd.21349

[28] Braegger CP, Ballabeni P, Rogler D, Vavricka SR, Friedt M, et al (2011) Epidemiology of Inflammatory Bowel Disease: Is There a Shift Towards Onset at a Younger Age? J Pediatr Gastr Nutr 53: 141–144. 10.1097/MPG.0b013e318218be35 21788753

